# The Process of Cessation Among Current Tobacco Smokers: A Cross-Sectional Data Analysis From 21 Countries, Global Adult Tobacco Survey, 2009–2013

**DOI:** 10.5888/pcd12.150146

**Published:** 2015-09-17

**Authors:** Lazarous Mbulo, Krishna M. Palipudi, Glenda Nelson-Blutcher, Komanduri S. Murty, Samira Asma, on behalf of the Global Adult Tobacco Survey Collaborative Group

**Affiliations:** Author Affiliations: Krishna M. Palipudi, Glenda Nelson-Blutcher, Samira Asma, Global Tobacco Control Branch, Office on Smoking and Health, National Center for Chronic Disease Prevention and Health Promotion, Centers for Disease Control and Prevention, Atlanta, Georgia; Komanduri S. Murty, Department of Behavioral Sciences, Fort Valley State University, Fort Valley, Georgia.

## Abstract

We analyzed data from the Global Adult Tobacco Survey (GATS) from 21 countries to categorize smokers by stages of cessation and highlight interventions that could be tailored to each stage. GATS is a nationally representative household survey that measures tobacco use and other key indicators by using a standardized protocol. The distribution of smokers into precontemplation, contemplation, and preparation stages varied by country. Using the stages of change model, each country can design and implement effective interventions suitable to its cultural, social, and economic situations to help smokers advance successfully through the stages of cessation.

## Objective

Tobacco use is a major cause of preventable diseases. Article 14 of the World Health Organization’s Framework Convention on Tobacco Control (FCTC) shows countries’ interest in implementing support for smoking cessation (1). However, to successfully implement Article 14, countries should identify the stages of cessation among tobacco smokers in their populations. This research attempts to identify those stages on the basis of the stages of change model so that countries can develop stage-specific interventions to help their smoker populations through successful cessation (2,3). Using Global Adult Tobacco Survey (GATS) data, we applied processes and stages of change to characterize the cessation status of smokers in 21 countries and discuss potential cessation interventions.

## Methods

GATS is a household survey of adults aged 15 years or older that uses a standardized protocol to collect data in all participating countries. A multistage cluster sampling design is used in all countries to achieve nationally representative samples (4). GATS was conducted from 2009 to 2013 in 21 countries: Argentina (2012), Bangladesh (2009), China (2010), Egypt (2009), Greece (2013), India (2010), Indonesia (2011), Malaysia (2011), Mexico (2009), Nigeria (2012), Panama (2013), Philippines (2009), Poland (2010), Qatar (2013), Romania (2011), Russia Federation (2009), Thailand (2011), Turkey (2012), Ukraine (2010), Uruguay (2009), and Vietnam (2010).

Response rates ranged from 65.1% (Poland) to 97.3% (Egypt), totaling 57,066 adult smokers from all 21 countries. All data (for each country) were weighted and poststratified to the national adult population in the respective countries (4). Details of GATS methods, including sampling design and data quality assurance, are available elsewhere (4).

GATS measures tobacco use and other key tobacco indicators. Current smokers were defined as adults who smoked either daily or less than daily. We focus on the first 3 stages of change (precontemplation, contemplation, and preparation), which were calculated by using responses to questions on last or most recent quit attempts in the past 12 months, duration of recent quit attempts, and future cessation intentions (2,5,6). The precontemplation stage included smokers who did not make an attempt to quit in the past 12 months and who did not consider quitting in the next 12 months. Although some studies considered only 6 months for intention to quit (2,5), GATS protocol used 12 months for this behavioral aspect. Contemplation included smokers who reported considering quitting within the next 12 months except for those considering quitting in the next month who had made a quit attempt of 24 hours or more in the past 12 months. The preparation stage included smokers who are thinking of quitting smoking within the next month and have made a quit attempt of at least 24 hours over the past 12 months (2,5,6).

## Results

Tobacco smoking prevalence among the 21 countries (Table), ranged from 3.9% (Nigeria) to 39.1% (Russia Federation). Across all countries, most smokers were in the precontemplation stage, followed by the contemplation stage and then the preparation stage. On average, 74.8% of smokers were categorized as being in the precontemplation stage, ranging from 61.4% (Qatar) and 61.6% (Bangladesh) to 89.5% (Indonesia).

**Table:** Weighted Estimates of Tobacco Smoking Prevalence^a^ and Stages of Cessation, by Country, Global Adult Tobacco Survey

Country	GATS Country Sample	Current Tobacco Smokers, % (95% CI)	Sample Size For Stages	Precontemplation,^b^ % (95% CI)	Contemplation,^c^ % (95% CI)	Preparation,^d^ % (95% CI)
Argentina 2012	6,645	22.1 (19.3–25.3)	1,648	75.4 (68.4–81.2)	16.2 (12.0–21.6)	8.4 (3.8–17.8)
Bangladesh 2009	9,629	23.0 (21.9–24.2)	2,217	61.6 (57.8–65.3)	26.3 (23.5–29.3)	12.1 (10.1–14.3)
China 2010	13,354	28.1 (26.7–29.7)	4,010	83.9 (80.3–87.0)	14.0 (11.2–17.4)	2.1 (1.4–3.1)
Egypt 2009	20,946	19.4 (18.8–20.1)	4,150	72.9 (70.7–75.0)	21.0 (19.1–22.9)	6.2 (5.1–7.4)
Greece 2013	4,359	38.2 (35.7–40.8)	1,664	86.2 (82.8–89.0)	13.2 (10.5–16.4)	0.7 (0.3–1.7)
India 2010	69,296	14.0 (13.4–14.6)	11,488	74.5 (72.7–76.3)	18.6 (17.1–20.1)	6.9 (6.0–7.9)
Indonesia 2011	8,994	34.8 (33.2–36.4)	2,853	89.5 (86.7–91.8)	7.1 (5.4–9.3)	3.3 (2.4–4.6)
Malaysia 2011	4,250	23.1 (21.2–25.2)	978	85.5 (81.3–88.8)	9.3 (6.6–13.0)	5.2 (3.5–7.6)
Mexico 2009	13,627	15.9 (14.8–17.1)	1,817	64.9 (61.8–67.9)	24.1 (21.4–27.0)	11.0 (9.1–13.2)
Nigeria 2012	9,765	3.9 (3.3–4.5)	424	63.8 (56.9–70.2)	23.3 (17.8–30.0)	12.9 (8.9–18.2)
Panama 2013	16,962	6.1 (4.9–7.5)	962	79.6 (73.2–84.9)	12.1 (7.4–19.0)	8.3 (5.8–11.8)
Philippines 2009	9,705	28.2 (27.0–29.5)	2,769	79.5 (77.3–81.5)	12.3 (10.7–14.1)	8.2 (7.0–9.7)
Poland 2010	7,840	30.3 (29.0–31.7)	2,416	68.5 (65.9–70.9)	24.2 (22.0–26.5)	7.3 (6.1–8.7)
Qatar 2013	8,571	12.1 (11.1–13.1)	1,073	61.4 (56.8–65.9)	31.2 (27.2–35.5)	7.4 (5.7–9.6)
Romania 2011	5,629	26.7 (25.0–28.4)	1,053	76.5 (73.7–79.2)	18.4 (16.0–21.1)	5.1 (4.0–6.4)
Russia Federation 2009	11,406	39.1 (37.8–40.5)	4,798	85.6 (83.7–87.4)	11.7 (10.1–13.6)	2.6 (2.1–3.4)
Thailand 2011	20,606	24.0 (22.8–25.1)	4,290	85.4 (83.4–87.2)	12.0 (10.3–13.9)	2.6 (2.0–3.5)
Turkey 2012	9,851	27.1 (25.8–28.3)	2,412	64.6 (61.8–67.3)	26.9 (24.6–29.4)	8.5 (7.2–10.0)
Ukraine 2010	8,173	28.9 (27.7–30.1)	2,392	74.1 (71.7–76.4)	20.7 (18.6–22.9)	5.3 (4.1–6.7)
Uruguay 2009	5,581	25.0 (23.3–26.6)	1,394	66.5 (63.1–69.7)	25.1 (22.2–28.3)	8.4 (6.5–10.7)
Vietnam 2010	9,925	23.8 (22.7–24.9)	2,258	70.8 (68.1–73.3)	21.6 (19.4–24.0)	7.6 (6.4–9.0)
Average		23.9	57,066^e^	74.8	18.5	6.7

Abbreviation: CI, confidence interval; GATS, Global Adult Tobacco Survey.

a Tobacco smoking prevalence is the proportion of adults who currently smoke tobacco daily or less than daily.

b Precontemplation included smokers who did not make an attempt to quit in the past 12 months and were not considering quitting in the next 12 months.

c Contemplation included smokers who consider quitting within the next month to 12 months except those considering quitting within next month who made a quit attempt of 24 hours or more in the past 12 months.

d Preparation included smokers who have made a quit attempt of 24 hours or more in the past 12 months and were considering quitting in the next month.

e Total number of smokers in the sample.

The percentage of smokers in the contemplation stage was lower than that of smokers in the precontemplation stage. Percentages ranged from 7.1% (Indonesia) to 31.2% (Qatar). The preparation stage yielded the lowest percentage of smokers in all countries at an average of 6.7%. Among the GATS countries, the percentage of smokers in the preparation stage ranged from less than 3.0% (China, Greece, Russia Federation, and Thailand) to 12.9% (Nigeria).

## Discussion

Distribution of smokers in stages of change varied by country. Six countries had more than 80% of smokers at the precontemplation stage, 8 countries had between 70% and 80%, and 7 countries had 60% to 69%. This variation may be attributable to 2 sets of factors: 1) environmental, social, economic, and culture conditions within each country (7); and 2) intercountry variations in adopting cessation interventions. For example, the 6 countries with a high proportion of smokers in precontemplation could consider adopting intervention strategies to target this population with specific goals to increase the awareness of the risk to self and others, the quality of life associated with cessation, and how the power of cessation is intrinsically motivated. Such intervention strategies should be developed within the population’s cultural milieu so that smokers in precontemplation will accept the messages and be motivated to consider quitting (3,6). Strategies may also include public education activities and media campaigns to motivate smokers at this stage to think of quitting (3,6). Similarly, adoption of strategies such as the use of graphic health warnings on cigarette packs and in conspicuous public places (eg, billboards) have increased smokers’ awareness of the health risks of smoking (8).

Consciousness raising and dramatic relief strategies, combined with recognizing the need to quit, making the decision to quit, learning to be a nonsmoker, and sustaining the quit attempt could also help motivate smokers to pass from the contemplation stage to the preparation stage (3,6,9). Thus, countries with a high proportion of smokers in the contemplation stage, such as Bangladesh, Qatar, Turkey, and Uruguay, could benefit from adopting both strategies to target this group.

Despite the low proportion of smokers in the preparation stage, countries should strategically target smokers at this stage to move them into action. For example, Bangladesh and Nigeria, each with more than 10% of smokers in the preparation stage, could adopt strategies that motivate this group to take action and avoid relapses into earlier stages. Processes of change strategies could include self-evaluation that allows smokers to reflect on their self-image in relation to their behavior (2,6). Use of health care providers with brief interventions or extensive intervention could be one strategy to help smokers reflect on their smoking behavior (10). Additionally, communities can organize “smoker cessation clubs,” wherein past smokers congregate and exchange their success stories, thereby influencing people in the stages of contemplation and precontemplation to move into the preparation stage.

We also found that countries with high smoking rates had high precontemplation rates, except for Panama, which is experiencing a decline in smoking rates but still has smokers who are having difficulty quitting (11). Particularly evident is the decline in the youth smoking rate from 13.2% (2002) to 4.3% (2008) (12).

Although the stages of change theory (13) has limitations, this study is the first to apply the model to data from 21 GATS countries and provide strategies anchored in experiential processes of the social cognitive model that help move smokers from precontemplation through action stages. Interventions suggested are broad in nature with built-in flexibility for each country to design interventions that are suitable to its culture, environment, and socioeconomic situation.

Study limitations also include the use of self-reported data, which is subject to possible recall or social desirability biases. Furthermore, differences in cessation stages across countries may reflect not only country-specific differences but also differences in survey timing. Overall, the stages of change model provides a useful framework for country-level cessation support, which remains critical to reduce tobacco use and its consequences.

## References

[1] World Health Organization. Framework Convention on Tobacco Control. Geneva (CH): World Health Organization; 2005. http://www.who.int/tobacco/framework/WHO_FCTC_english.pdf. Accessed May 8, 2015.

[2] Velicer WF , Prochaska JO , Fava JL , Rossi JS , Redding CA , Laforge RG , Using the transtheoretical model for population-based approaches to health promotion and disease prevention. Homeost Health Dis 2000;40:174–95.

[3] Prochaska JO , Redding CA , Evers KE . The transtheoretical model and stages of change. In: Glanz K, Marcus Lewis F, Rimer BK, editors. Health behavior and health education: theory, research and practice. San Francisco (CA): Jossey-Bass; 2002. p. 60–84.

[4] Global Adult Tobacco Survey Collaborative Group. Global adult tobacco surveys (GATS): sample design manual. Atlanta (GA): Centers for Disease Control and Prevention; 2010.

[5] Etter JF , Perneger TV , Ronchi A . Distributions of smokers by stage: international comparison and association with smoking prevalence. Prev Med 1997;26(4):580–5. 10.1006/pmed.1997.0179 9245682

[6] DiClemente CC , Prochaska JO , Fairhurst SK , Velicer WF , Velasquez MM , Rossi JS . The process of smoking cessation: an analysis of precontemplation, contemplation, and preparation stages of change. J Consult Clin Psychol 1991;59(2):295–304. 10.1037/0022-006X.59.2.295 2030191

[7] Dotinga A , Schrijvers CT , Voorham AJ , Mackenbach JP . Correlates of stages of change of smoking among inhabitants of deprived neighbourhoods. Eur J Public Health 2005;15(2):152–9. 10.1093/eurpub/cki112 15941760

[8] Shanahan P , Elliott D . Evaluation of the effectiveness of the graphic health warnings on tobacco product packaging 2008. Canberra (AU): Australian Government Department of Health and Ageing; 2009.

[9] Brown JM . Redefining smoking and the self as a nonsmoker. West J Nurs Res 1996;18(4):414–28. 10.1177/019394599601800404 8797366

[10] Hudmon KS , Benger BA . Pharmacy applications of the transtheoretical model in smoking cessation. Am J Health Syst Pharm 1995;52(3):282–7. 7749955 10.1093/ajhp/52.3.282

[11] Burns DM , Warner KE . Chapter 1. Smokers who have not quit: is cessation more difficult and should we change our strategies. In: Ruppert W, Amacher RH, Marcus SE, Shopland DR, editors. Those who continue to smoke: is achieving abstinence harder and do we need to change our interventions? Smoking and tobacco control monograph no. 15. Bethesda (MD): US Department of Health and Human Services, Public Health Service, National Institutes of Health, National Cancer Institute; NIH Pub. No. 03-5370, September 2003. p. 101–25.

[12] Centers for Disease Control and Prevention. Changes in tobacco use among youths aged 13–15 years — Panama, 2002 and 2008. MMWR Morb Mortal Wkly Rep 2009;57(53):1416–9. 19129746

[13] Whitelaw S , Baldwin S , Bunton R , Flynn D . The status of evidence and outcomes in stages of change research. Health Educ Res 2000;15(6):707–18. 11142078 10.1093/her/15.6.707

